# Photobiomodulation versus fractional carbon dioxide laser for stria alba in phototype III-IV: a randomized controlled study

**DOI:** 10.1007/s10103-024-04107-x

**Published:** 2024-06-19

**Authors:** Vanessa Hafez, Doaa Mahgoub, Elsayed Mohamed Ali Satour, Marina Mozeih Shaker Mikhail, Mona El-Kalioby

**Affiliations:** 1 Department of Dermatology, Kasr Al-Ainy Faculty of Medicine, Cairo University, Cairo, Egypt; 2 Department of Basic Science, Faculty of Physical Therapy, Pharos University, Cairo, Egypt

**Keywords:** Fractional carbon dioxide laser, Low level light therapy, Infra-red diode 808 nm, Photobiomodulation, Striae distensae

## Abstract

Striae distensae are common dermatological complaint. Cold laser using low-level light/laser therapy (LLLT) offers healing and analgesic effects and was not yet compared to ‘hot lasers’ efficacy. Study objective: to assess the efficacy and safety of LLLT in the management of stria alba compared to fractional carbon dioxide (FCO_2_) laser alone and to the combined use of both devices. Thirty patients with stria alba were randomized to receive either LLLT using diode 808 nm; 8–12 sessions, 2–3 sessions weekly (Group A) or FCO_2_ laser; 2 monthly sessions (Group B) or combined both devices simultaneously (Group C). Follow up was at 1 month and 3 months after last session. The efficacy of LLLT was statistically comparable to FCO2, despite numerical superiority of the latter. The combined group had the least numerical values in all efficacy outcomes. Patients in LLLT group did not experience any downtime. LLLT is effective in the management of stria alba comparable to the FCO_2_ laser. The lack of downtime with LLLT is reflected positively on patient’s satisfaction. However, this is counterbalanced by the frequent weekly visits. Although adding LLLT to FCO2 laser palliates the laser side effects but it offers the least efficacy. **Trial registration number** NCT04165226 (clinicaltrials.gov)

## Introduction

Striae distensae (SD), also known as stretch marks, are common dermatological complaint that can cause great distress to patients regardless of any medical implications [1]. The pathogenesis of SD involves changes in the extracellular matrix; decreased secretion of mucopolysaccharides, alterations in the elastic and collagen fibers in response to various factors such as fluctuation of steroid hormones, obesity, pregnancy, etc [2].

Several tools have been proposed to correct this disfiguring condition for patients seeking treatment. None to date reached alone high enough satisfactory results to be considered as a cornerstone of treatment. Research advises the combination of modalities for better results. Available modalities include lasers, fractional radiofrequency, microneedling, dermabrasion, chemical peeling. Topical agents have limited efficacy. Among lasers, fractional carbon dioxide (FCO2) was found to yield better results in comparison to other lasers (pulsed dye, NDYAG, diode, excimer and copper bromide). The higher the parameters, the better the results of FCO2 laser, but also the higher the incidence of downtime, procedural pain, prolonged erythema and post-inflammatory hyperpigmentation, particularly the darker skin types [3].

The application of photobiomodulation using LLLT also called ‘cold laser’ is booming in the last decades for variable indications due to its regenerative, anti-inflammatory, and analgesic properties [4, 5, 6, 7]. We hypothesized a beneficial effect of LLLT on stria without the drawbacks of FCO2 laser. Even more, the combination of both was expected to yield synergistic efficacy along with reduced side effects of the latter. This work aimed to assess the efficacy and safety of infra-red diode 808 nm in the treatment of stria alba in comparison to FCO_2_ alone and to combined both therapies.

## Patients and methods

### Study design

This study was designed as a randomized, controlled, clinical trial with three 1:1:1 interventional arms. The study protocol, published in the clinicaltrials.gov registry (NCT04165226), conformed to the ethical guidelines of the 1975 Declaration of Helsinki and was approved by the Dermatology research ethical committee (DermaREC) of the Faculty of Medicine, Cairo University. The study report follows the CONSORT guidelines for reporting randomized controlled clinical trials [8].

### Patients

Patients were recruited from the dermatology outpatient clinic at Kasr Al-Ainy Faculty of Medicine, Cairo University. Healthy subjects of both genders, aged above 18, with Fitzpatrick’s skin phototype III-IV, presenting with striae alba were eligible for the study. Stria alba affecting any body area and of any duration were included. Exclusion criteria included stria rubra, using topical/systemic corticosteroids, topical/systemic retinoid, vitamin C, or vitamin E within 3 months, performing any procedure for striae (laser, radiofrequency, dermabrasion, microdermabrasion or chemical peeling) within 6 months, patients with connective tissue diseases, presence of skin infection, or any disease that affects the wound-healing process, previous history of hypertrophic scar, keloid, cancers or immunosuppression, and current pregnancy or lactation.

All patients signed an informed written consent for participation and photography. A thorough history was taken from all patients. An examination was done to determine the type of striae, their anatomical distribution and the possible precipitating factors.

The patients were randomized into one of three interventional groups based on a computer-generated list (prepared by an independent colleague), either LLLT (Group A), FCO_2_ (Group B), or a combination of FCO_2_ laser and LLLT (Group C). Allocation to groups was done using concealed envelopes.

### Interventions

#### Group A (LLLT)

HPL Pagani Diode 808/915nm LLLT 3.2 W (Fimad Elettromedicali SRL®, Catanzaro, Italy) was used in the Physiotherapy and rehabilitation department, New Kasr Al Ainy hospital. The patients were offered 12 sessions of the low-level diode laser 808 nm with 2–3 sessions weekly for 4–6 weeks. Based on investigator’s experience with LLLT, a decision was made that patients who completed a minimum of 8 sessions were included in data analysis. The parameters were adjusted individually according to the surface area to be treated with the optimum dose of 5–12 joules/ cm^2^, continuous non-contact beam by scanning criteria at a 50 cm optimum distance.

#### Group B (FCO_2_)

Each patient received two monthly sessions of Fractional CO_2_ laser (FIRE-XEL ablative device from Bison Medical®, Seoul, South Korea). A single pass was done at energy level 12–15 mJ, pulse width 800–1000 µs, overlap 1, density level 1, stack 1. The probe was usually adjusted to treat the stria only. Whenever there was a confluence of the stria, the whole area was treated including normal skin in between.

#### Group C (combined FCO_2_ laser and LLLT)

The patients were treated with both treatment modalities, where they had monthly FCO_2_ sessions followed 5 days later with LLLT sessions, 2–3 sessions/week for 8–12 sessions.

### Outcomes

The treated areas were photographed (in standardized settings) using a Samsung S7 edge digital camera (12 megapixels) on days 1, 30, 60, 90 and 120. Efficacy and safety outcomes were assessed 1 and 3 months after the last session by 1 unblinded physician and 1 blinded physician who evaluated patient’s photographs. Efficacy was assessed using *Physician/Patient’s global assessment of improvement (GAI)* (0 = worsened, 1 = minimal improvement or steady state (< 25%), 2 = moderate improvement (26 − 50%), 3 = marked improvement (51 -75%), 4 = near total improvement (> 76%)) and *patient satisfaction score* (A = not satisfied, B = somehow satisfied, C = highly satisfied). Treatment success was defined as improvement ≥ 50% in patients’ and\or physician’s GAI (marked/near total improvement).

The number and duration (in days) of adverse events were recorded, particularly pain, erythema, edema, and crusting. The occurrence of secondary infection and post-inflammatory hyper-pigmentation were also monitored.

### Statistical methods

The sample size was calculated using G-Power 3.1.9.2, based on data collected in a pilot study with 9 subjects (three in each group). A sample size of 7 patients per arm was calculated to provide 95% power and a 0.05 type 1 error rate, with a weighted standard deviation of 0.95. To account for the estimated sample loss rate, 30 patients must be included in the study, randomly assigned and equally distributed to one of the three groups.

Data were summarized using mean, standard deviation, median, minimum, and maximum in quantitative data and using frequency (count) and relative frequency (percentage) for categorical data. Comparison between quantitative variables were done using the non-parametric Kruskal-Wallis with adjusted Mann-Whitney as post-hoc test. Chi-square (χ2) test was used for comparing categorical data. Exact test was used instead when the expected frequency was less than 5. P-values less than 0.05 were considered statistically significant. Per-protocol analysis was used for efficacy outcomes. Crude change in outcomes for both groups from baseline to post-treatment and follow up was provided as mean change scores with standard deviations or proportions. Treatment success was calculated on an intention-to-treat basis (= number and percent of patients achieving treatment success out of total number of patients, including drop-outs).

## Results

**Fig. 1:**
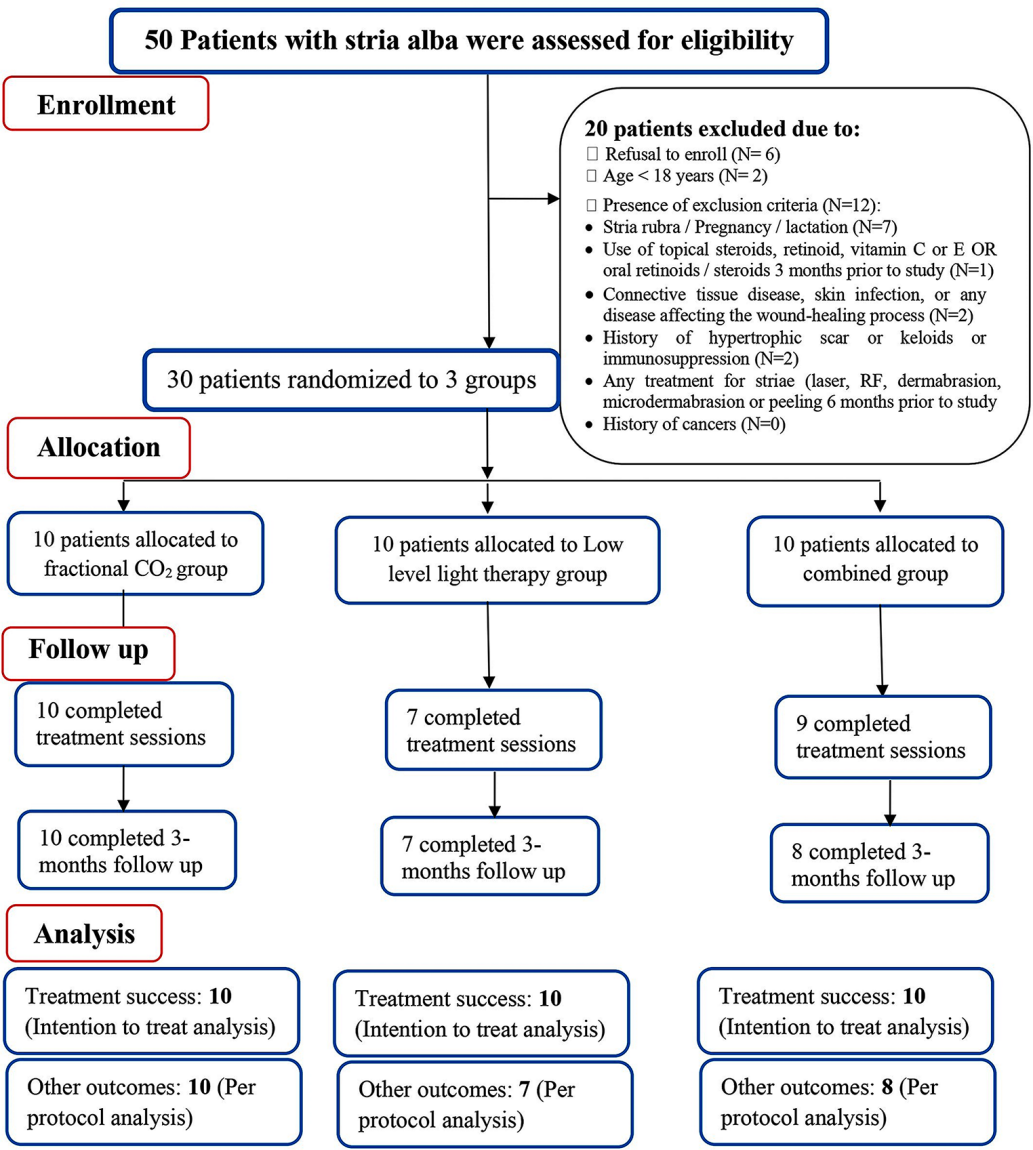
Patient’s flow diagram according to CONSORT guidelines for reporting randomized controlled trials

The patients’ flow diagram is shown in Fig. 1.

### Baseline characteristics of patients

Thirty patients were enrolled, including 1 (3.3%) male and 29 (96.7%) females. Ages ranged between 18 and 41 years with a mean of 27.63 ± 6.061. Eleven (36.7%) patients had skin phototype III while the other 19 (63.3%) patients had skin phototype IV. The mean duration of the stria was 6.93 ± 4.54 years. All groups had matching characteristics at baseline except for gender. Twenty-six out of 30 patients completed the study.

### Comparison of the three groups after treatment

#### Effectiveness outcomes

**Fig. 2:**
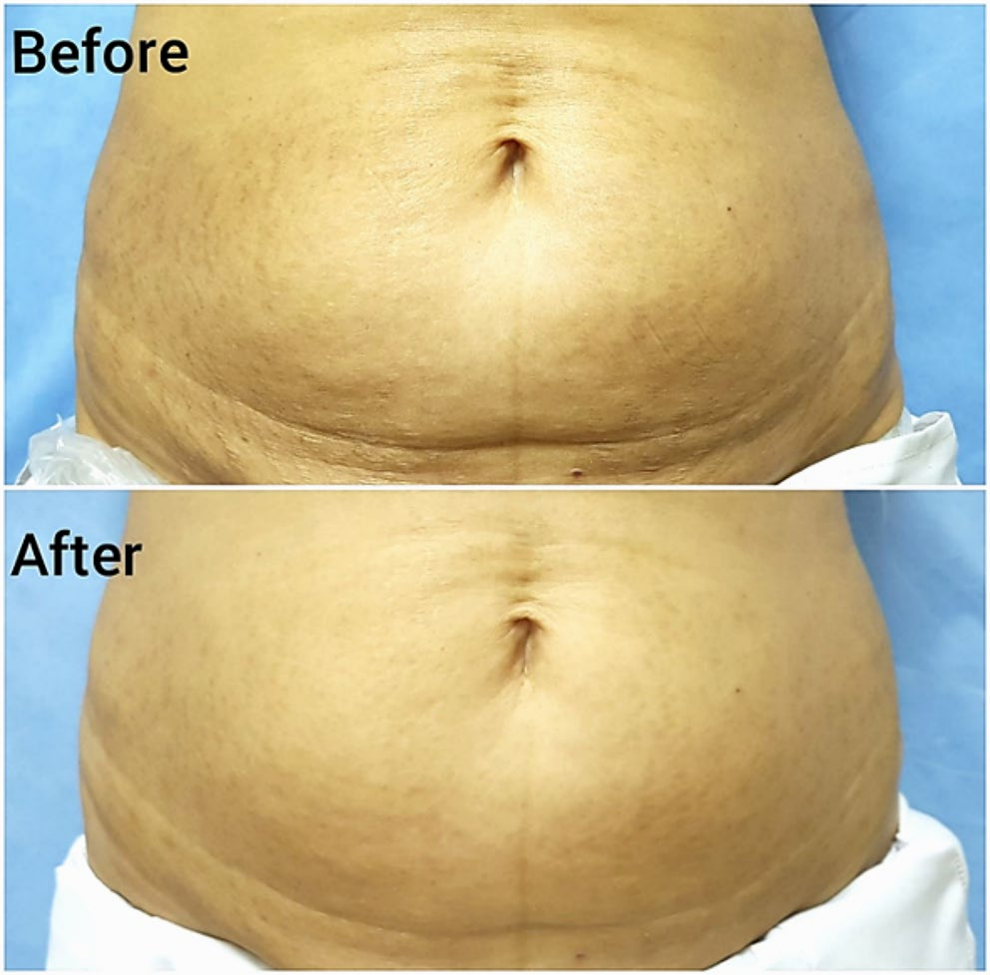
Stria distensae, a representative patient treated with low level light therapy (diode 808 nm). Moderate response is seen after 3 months from the end of treatment (Patient global assessment of improvement = 40%, physician global assessment of improvement = 47.5%, patient satisfaction score = 50%)

**Fig. 3:**
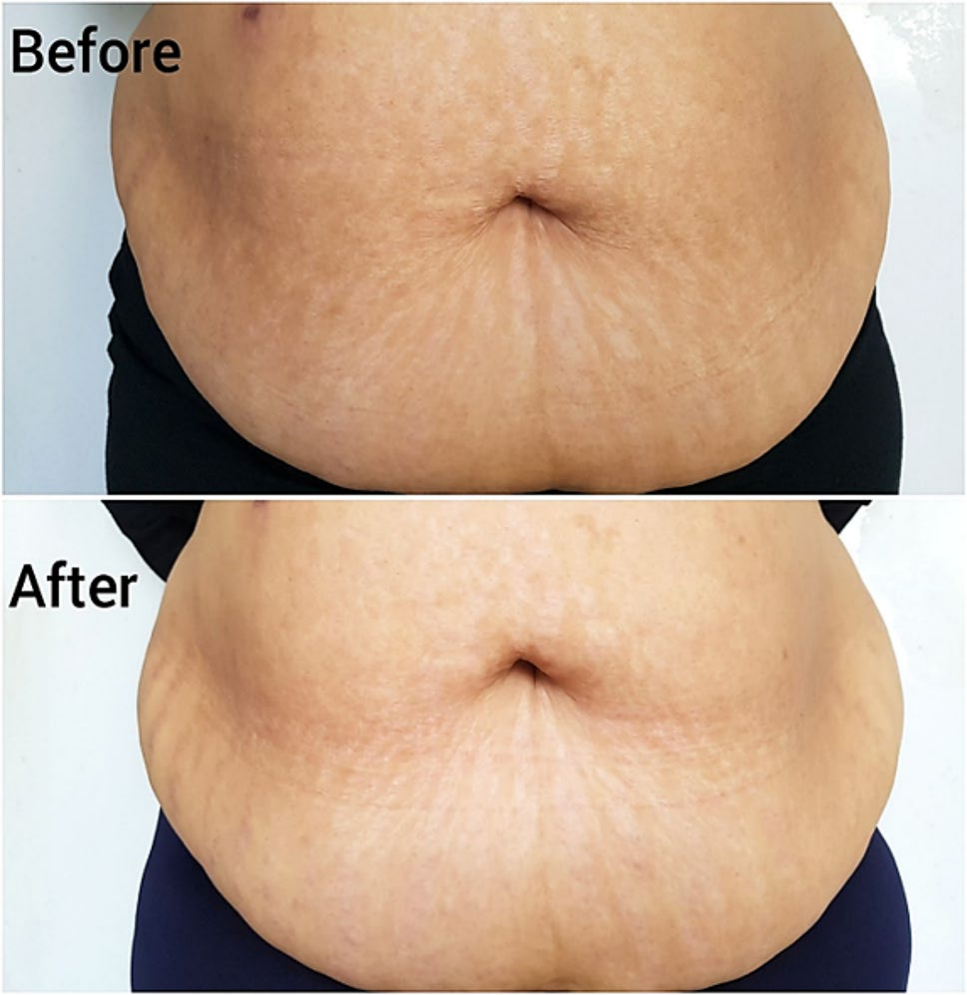
Stria distensae, a representative patient treated with fractional carbon dioxide laser. Excellent response is seen after 3 months from the end of treatment. (Patient / physician global assessment of improvement = 85%, patient satisfaction score = 90%)

**Fig. 4:**
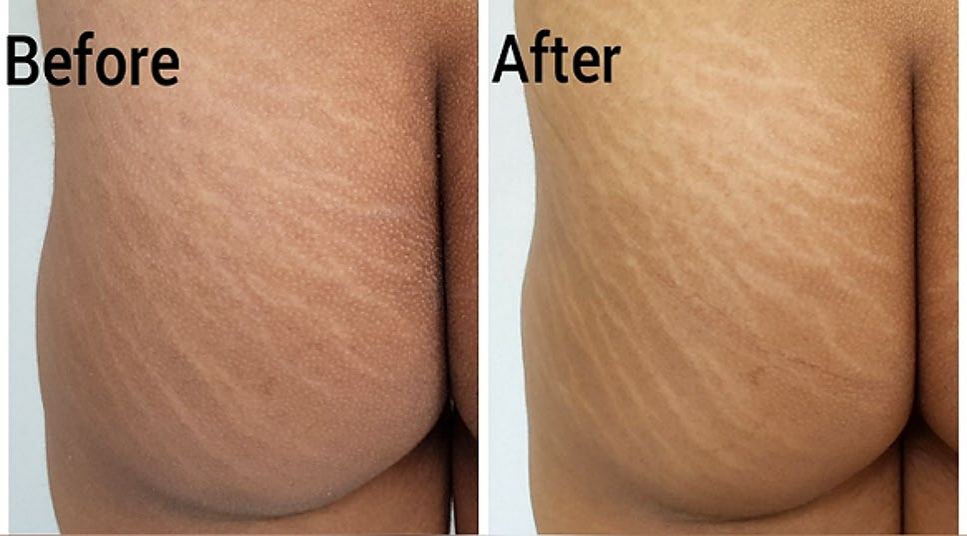
Stria distensae, a representative patient treated with combined low level light therapy (diode 808 nm) and fractional carbon dioxide laser. Poor response is seen after 3 months from the end of treatment (Patient global assessment of improvement = 20%, physician global assessment of improvement = 7.5%, patient satisfaction score = 20%)

The efficacy of LLLT was comparable to FCO2 regarding patient GAI, physician GAI and patient satisfaction scores after 1 and 3 months. The combined group showed significantly lower efficacy compared to FCO2 regarding patient GAI, physician GAI and patient satisfaction and to LLLT regarding satisfaction score after 3 months (Table 1; Figs. 2, 3 and 4). Based on the treatment success definition, the combined group was significantly less effective than both other groups, while LLLT and FCO2 showed comparable treatment success.

**Table 1:** Comparison of the efficacy outcomes between the 3 interventional arms after 1 and 3 months of therapy (M1, M3)

	Group A: LLLT (*n* = 10)	Group B: FCO_2_ laser (*n* = 7)	Group C: Combined (*n* = 8)	*p*-value A vs. B	*p*-value A vs. C	*p*-value B vs. C
	Median (range)					
*Patient global assessment of improvement*						
M1 Patient GAI (%)	40 (30–80)	65 (20–80)	20 (20–60)	0.887	0.055	0.017*
M3 Patient GAI (%)	40 (30–80)	70 (20–90)	30 (10 − 8)	0.161	0.055	0.01*
*Physician global assessment of improvement*						
M1 Physician GAI (%)	45 (30–75)	60 (20–80)	30 (15–55)	0.536	0.114	0.133
M3 Physician GAI (%)	45 (20–82.5)	61.25 (17.5–92.5)	45 (5–50)	0.27	0.606	0.035*
*Patient satisfaction score*						
M1 Patient satisfaction score %	50 (30–80)	75 (20–85)	30 (20–65)	0.364	0.071	0.008*
M3 Patient satisfaction score (%)	50 (40–90)	77.5 (20–90)	40 (10–80)	0.27	0.012*	0.01*
*Treatment success (intention to treat analysis)*						
Patient GAI	3/10	8/10	1/10			
Physician GAI	2/10	7/10	2/10			
Patient satisfaction	5/10	8/10	2/10			

FCO2: Fractional CO laser, LLLT: Low level light therapy, GAI: Global assessment of improvement, * p value is significant if ≤ 0.05

#### Safety outcomes

The patients in LLLT did not develop any side effects during the first and second months of therapy. The combined group had less incidence and decreased duration of side effects (edema, pain, erythema, itching and peeling) in comparison to FCO2 after the first and second sessions, although the difference was not significant (Tables 2 and 3).

**Table 2:** Comparison between the occurrence of adverse events [number (%)] between the 3 interventional arms during the first and second month of therapy

	During first month				During 2nd month			
	Group A LLLT (*n* = 10)	Group B FCO_2_ (*n* = 7)	Group C combined (*n* = 8)	*p*-value B vs. C	Group A LLLT (*n* = 10)	Group B FCO_2_ (*n* = 7)	Group C combined (*n* = 8)	*p*-value B vs. C
Edema	0 (0)	8 (80)	9 (100)	0.474	0 (0)	10 (100)	7 (87.5)	0.444
Pain	0 (0)	10 (100)	9 (100)	-----	0 (0)	10 (100)	6 (75)	0.183
Erythema	0 (0)	9 (90)	9 (100)	1	0 (0)	10 (100)	8 (100)	----
Itching	0 (0)	9 (90)	8 (88.9)	1	0 (0)	9 (9)	7 (87.5)	1
Peeling	0 (0)	10 (100)	8 (88.9)	0.474	0 (0)	10 (100)	8 (100)	----

FCO2: Fractional CO laser, LLLT: Low level light therapy

**Table 3:** Comparison of the median duration of side effects (in days) between Group B and C after first and second session of FCO2 laser

After first session				After second session		
	Group B FCO2 (*n* = 7)	Group C combined (*n* = 8)	*p*-value	Group B FCO2 (*n* = 7)	Group C combined (*n* = 8)	*p*-value
Edema	1.5	1	0.692	2	1.5	0.256
Pain	1.5	1	0.461	2	1	0.096
Erythema	3.5	2	0.103	3.5	2.5	0.318
Itching	1	1	0.829	3.5	2	0.618
Peeling	10.5	7	0.068	8	7	0.29

FCO2: Fractional CO laser

Persistent post-inflammatory hyperpigmentation after the first session of FCO_2_ was reported by one patient only from the combined group, which discouraged her from performing the second session.

### LLLT parameters

The parameters were adjusted individually according to the surface area to be treated with the optimum dose 5–12 J/cm2. The median area on which the LLLT was applied was 269 cm^2^ in the LLLT group and 169 cm^2^ in the combined group. The median intensity used for the LLLT group was 7 J/cm2 as compared to 8 J/cm2 in the combined group. The median time applied during the session for the LLLT group patients was 14.27 min versus 10.01 min in the combined group.

## Discussion

To the best of our knowledge, this work is the first to compare LLLT efficacy and safety to FCO2 and to combined both modalities in stria alba. The results of the present study show that LLLT is comparably effective to FCO2 in the management of stria alba in phototypes III-IV with an added benefit of lack of discomfort and downtime that are usually reported with FCO_2_ therapy. However, LLLT requires more frequent sessions, at least biweekly, for a minimum of 8 and a maximum of 12 sessions. The combination of both modalities, against our expectations, offers the least efficacy, but it also decreases the downtime of FCO_2_ laser (namely: erythema, edema, pigmentation, itching or peeling), although the difference does not achieve statistical significance.

The efficacy of LLLT, FCO2 and their superiority over combined both modalities was evidenced by the median patient GAI at three months after treatment (40%, 70% and 30% respectively), the median physician GAI (45%, 61.25% and 45% respectively) and the patient satisfaction score (50%, 77.5% and 40% respectively). Similarly, the intention-to-treat analysis for treatment success showed statistically comparable results between both LLLT and FCO2 groups, with again, better numerical values in the FCO2 group and the least numerical values in the combined group: marked to near-total improvement was reported by 30%, 80% and 10% of patients respectively and was documented by physicians in 20%, 70% and 20% of patients respectively, and highly satisfied patients were 50%, 80% and 20% of the patients respectively.

Two Brazilian comparative studies assessed the efficacy and safety of LLLT in the treatment of striae alba and reported significant improvement of the striae at one month after therapy in response to 660 nm [9], and less response to 830 nm in comparison to 660 nm [10], with no side effects reported [9, 10]. Both studies used Laserpulse (Ibramed®, Amparo, Brazil) adjusted at 30mW power, 4 J/cm^2^ energy density for 12 sessions over 4 weeks and used photographs with digital planimetry for quantification of surface area of stria. In fact, in vitro studies showed that, although both wavelengths are involved in wound healing, fibroblasts, the primary cell for collagen synthesis, respond better to 633 nm light, while the 833 nm light stimulates better the other cells of wound healing process (mast cells, neutrophils, and macrophages) [11]. This might explain why the LLLT arm using 808 nm diode in our study showed modest efficacy, and better results were demonstrated in the Brazilian studies by using the 660 nm LLLT in comparison to 830 nm [9, 10]. The beneficial effect of LLLT on wound healing was demonstrated by several in vitro and in vivo studies where positive actions were shown in tissue perfusion and stimulation to neovascularization, fibroblastic proliferation and keratinocytes, increased synthesis and deposition of collagen and hydroxyproline, decrease of inflammatory mediators such as IL-1β, IL-10, TNF-α and NF-κB, decreased stress oxidation and acceleration of tissue healing [10]. Both studies praised the convenience of LLLT as a safe and painless tool, as we could also demonstrate in our study.

The efficacy of the LLLT drastically depends on the use of the correct parameters. The Hormesis phenomenon states that too low or too high doses may result in insignificant effect or even inhibitory action. Therefore, adjusting the dose of LLLT application needs special attention [12]. Nevertheless, the dosing of LLLT for different indications is till now a matter of uncertainty [13].

Unlike LLLT, there are numerous reports about FCO_2_ in the treatment of stria alba; showing evidence for its efficacy clinically and histologically [13–22]. H&E and Masson-trichrome staining of biopsies showed greater average epidermal and dermal thickness after treatment of striae by FCO_2_ laser [14]. The number of sessions in these studies ranged from 4 to 5, the parameters used varied according to the device and patients’ skin phototype. The number of cases showing ≥ 50% improvement after 4 to 5 sessions varied between 20 and 47% [17–23].

A retrospective case series in Iran [22] studied the effect of four sessions of FCO2 (microxel MX700, Korea) in 24 female patients with skin type II-IV with the following parameters according to the skin type; frequency: 1000 Hz, duration: 130–200 s and energy: 40–60 J/cm2. Photos were obtained one month after the last treatment, and the authors reported 16.7% minimal improvement (in contrast to 20% in the fractional group in our study), 54.2% moderate improvement (in contrast to 10% in our study), 29.2% marked improvement (in contrast to 40% in our study), and no patients showed near total improvement (in contrast to 30% in our study), all based on physician’s global assessment of improvement.

Taking into consideration that the results of two sessions of FCO_2_ laser of the present study are comparable to those achieved by four sessions on similar skin phototype [22], with even higher percent of near-total improvement in the present study, the authors are confident that the dose regimen applied in the present study is a successful one to be recommended: combining a long pulse duration (800-1000us) with a moderate spacing (1 mm interdot) aimed at proper heating of the dermis for stimulation of neocollagenesis, while preserving the inter-dot tissue to allow for faster regeneration.

As regards the safety outcomes, the patients in the LLLT group experienced higher tolerability, as none of the patients suffered from side effects after the sessions, while the side effects after the FCO_2_ sessions included pain, during and after the procedure, edema, erythema, itching and peeling for variable durations each. This might explain the lack of dropouts in the LLLT group compared to the other 2 groups. Post-inflammatory hyperpigmentation that persisted after the first session of FCO_2_ group was reported by one patient from the combined group.

We hypothesized that the anti-inflammatory and analgesic effect of LLLT would palliate the side effects of FCO_2_ laser. Indeed, patients in the combined group showed a modestly shorter duration of the FCO_2_ reported side effects, in comparison to FCO_2_ alone group, although not reaching statistical significance. A cumulative anti-inflammatory effect along sessions was not observed in the combined group, as evidenced by the relative similarity of the duration of side effects between the first and second sessions of FCO_2_. This suggests that starting the LLLT earlier before the FCO_2_ laser session, instead of immediately after, would have probably not added more benefit. However, we suggest that the anti-inflammatory effect of LLLT was responsible for the dampened efficacy of FCO_2_ laser in the combined arm, where the least efficacy was recorded. The mechanism of action of FCO_2_ laser in management of stria alba is based on the formation of micro-thermal zones of injury leading to an inflammatory response, inducing the expression of heat shock protein 70 and type 1 procollagen by dermal dendritic cells along with enhancement of the antioxidant enzymes which induces the production of new collagen [16, 23].

Interestingly, 4 patients who underwent the LLLT reported at the EOS an unwanted pregnancy event, with two patients reporting previous history of infertility. This raises the question whether LLLT applied on the abdomen causes ovarian stimulation, and if confirmed such response might be of use in infertility issues but it suggests that unwanted pregnancy might be a warning to include in patients consent before receiving LLLT, particularly if performed over the abdomen and pelvis. It is known that LLLT may be contraindicated in cancer patients due to its hypothesized effect to stimulate the growth of cancer cells in cell cultures [24], but it is still unclear if there is a universal stimulatory growth effect on the whole body that contributed to the pregnancy of the four participants. This issue deserves further investigation.

This study is limited by its small number of participants, the lack of tissue samples to provide histopathological evidence of response, and, as data is sparse about the best parameters for LLLT in skin applications, the parameters used in this study were based on the investigator’s personal experience.

In conclusion, this randomized controlled trial shows that LLLT using Diode 808 nm is effective and safe in management of stria alba, comparable to FCO_2_ laser, in skin type III-IV. The lack of discomfort and downtime in the LLLT group reflected on patient’s satisfaction positively in disproportion to its modest efficacy. However, this is counterbalanced by the obligation to submit to frequent weekly visits for 8 to 12 sessions. The combination of both modalities, against our expectations, offers the least efficacy, but palliates the side effects induced by the FCO_2_, by shortening their duration. The systemic biostimulatory effect of the sessions can also be an advantage in favor of LLLT, as well as the anti-inflammatory and analgesic effect of LLLT that can be used as an adjuvant to other therapeutic modalities in cosmetology, provided that this modality does not depend on the wound response. Further studies are needed to define the adequate dosing regimens of LLLT in dermatological applications.
